# Ensuring that the Improving Access to Psychological Therapies (IAPT) programme does what it says on the tin

**DOI:** 10.1111/bjc.12264

**Published:** 2020-08-16

**Authors:** Michael J. Scott

**Affiliations:** ^1^ Psychological Therapies Unit Liverpool UK

I welcome the opportunity to comment on the recent paper ‘Improving Access to Psychological Therapies (IAPT) in the United Kingdom: A systematic review and meta‐analysis of 10‐years of practice‐based evidence’ by Wakefield *et al*. (2020) published in the Journal. This paper is of considerable importance because the authors conclude that IAPT’s results approach the 50% recovery rate found in randomized controlled trials (RCTs) of cognitive behaviour therapy for depression and the anxiety disorders. Taken at face value, this meta‐analysis provides justification for current IAPT services, which have cost the taxpayer £4 billion. Further the study could fuel the funding of not only the provision of services to more clients but accelerate the expansion of IAPT services without any diagnostic boundary. There can be no doubt that improving access to psychological therapies is an important societal aim, as only a significant minority of those with mental health problems are beneficiaries. But this is a far cry from legitimizing the tangible expression of this goal in the guise of the IAPT service.

## Allegiance bias and real‐world outcome

In the Wakefield *et al*. (2020) paper all the authors declare ‘no conflict of interest’. But the corresponding author of the study, Stephen Kellett, is an IAPT Programme Director. The study is therefore open to a charge of allegiance bias. It is therefore not surprising that Wakefield *et al*. (2020) fail to make the distinction between IAPT’s studies and IAPT studies. By definition, the former have a vested interest, akin to drug manufacturer espousing the virtues of its psychotropic drug. Whilst an IAPT study is conducted by a body or individual without a vested interest, in this connection Wakefield *et al*. (2020) have implicitly misclassified this author’s IAPT study, Scott (2018). In their study, Wakefield *et al*. (2020) make reference to the Scott (2018) study with a focus on a subsample of 29 clients (from the 90 IAPT clients) for whom psychometric test results were available in the GP records. But in Scott (2018) it was made clear that concluding anything from such a subsample was extremely hazardous. The bigger picture was that 90 IAPT clients were independently assessed using a ‘gold standard’ diagnostic interview, either before or after their personal injury (PI) claim. Independent of their PI status, it was found that only the tip of the iceberg lost their diagnostic status as a result of IAPT treatment. Wakefield *et al*. (2020) were strangely mute on this point. They similarly failed to acknowledge that the ‘IAPT’s studies’ involved no independent assessment of IAPT client’s functioning and there was no use of a ‘gold standard’ diagnostic interview.

## The failure to demonstrate an added value

The Wakefield *et al*. (2020) study did not include a comparison of IAPT’s claimed outcomes with an appropriate counterfactual. For a new service to warrant continued funding, it must demonstrate that it is better than if the service never existed. But data from psychological services that pre‐dated IAPT suggest that IAPT has conferred no added value. In 2006, Mullin *et al*. (2006) examined the effects of counselling/therapy in more than 11,000 clients and concluded that between 5 and 6 clients out of every 10 met the criterion for recovery. These authors used the same criterion with regard to the reliable change index (Jacobsen & Truax, 1991) as used by IAPT, but used the CORE‐OM self‐report measure (Barkham *et al*., 2006) rather than the PHQ9 (Kroenke *et al*., 2001)/GAD7 (Spitzer *et al*., 2006).

## IAPT’s studies fail to clear methodological bars for evidence supported treatments (ESTS)

The past decade has witnessed a refinement in the criteria necessary for psychological interventions to be regarded as evidence supported. This has included (1) more detailed examination of the risk of bias (Higgins *et al*., 2011) (2) the need for comparisons with active control conditions (Carpenter *et al*., 2018; Giudi *et al*., 2018), (3) the need for independent blind assessment, a combination of observer and patient‐reported outcome measures together with a determination of the duration of recovery (Giudi *et al*., 2018), and (4) the need for measures of treatment fidelity and the need to test out a supposed EST in real‐world settings with evaluators independent of those who developed the protocols (Tolin *et al*., 2015). In the same decade, IAPT has greatly expanded, but Wakefield *et al*. (2020) fail to acknowledge that IAPT’s studies are largely invalidated by these considerations. These authors seem unaware of a highering of the methodological bar, for interventions to be regarded as ESTs.

## IAPT’s treatment infidelity

As Wakefield *et al*. (2020) acknowledge, IAPT does not utilize any measure of treatment fidelity, but appear not to appreciate the gravity of this. IAPT espouses NICE approved treatments but there can no certainty that this is delivered at the coal face, for example, that an IAPT clinicians claimed trauma focussed CBT (TFCBT) actually took place. Further IAPT eskews diagnosis (National Collaborating Centre for Mental Health, IAPT Manual, 2019), making it impossible to compare the effectiveness of TFCBT delivered in the service with the efficacy found in randomized controlled trial (RCT) of PTSD studies where TFCBT was employed.

## Dubious points of reference

More generally, IAPT’s client population is so heterogeneous that no meaningful comparisons can made with the results of RCTs. A similar criticism can be made of the Stewart and Chambless (2009) meta‐analysis of effective studies which Wakefield *et al*. (2020) used as a reference point for IAPT’s studies. For example, the Stewart and Chambless (2009) study cites the Westbrook and Kirk (2005) effectiveness study when making comparison with RCTs in efficacy studies, but the Westbrook and Kirk (2005) population were of unknown diagnostic status, making for a doubtful comparison. Wakefield *et al*. (2020) also cite a study by Thimm and Antonsen (2014) as a reference point for their IAPT’s studies, but the former used a standardized diagnostic interview to establish that their clients were depressed no such yardstick has been employed by IAPT, making it uncertain whether IAPT’s claimed depressed clients are comparable to those in this study. Comparison is doubly problematic as the focus in the Thimm and Antonsen (2014) study was exclusively on those who underwent group CBT.

## IAPT’s studies are of completers, despite most clients dropping out

Whilst Wakefield *et al*. (2020) acknowledge the importance of an intention to treat analysis, they fail to highlight how the absence of this undermines their review. IAPT’s studies focus primarily on completers, defined as attending two or more sessions. Notwithstanding this strange definition of completion, the rate of dropout was 62.5%, Richards and Borglin (2011). This pattern of engagement is identical to that found in Scott (2018). Given that clients completing treatment in IAPT are atypical, the conclusions of Wakefield *et al*. (2020) are of doubtful real‐world significance. Wakefield *et al*. (2020) seek to buttress the status of IAPT’s studies by making comparison with the Stewart and Chambless (2009) meta‐analysis, but 52 of the 56 studies cited in the latter refer to completer studies. This is an inappropriate yardstick given the ‘haemorrhaging’ of IAPT clients reported by Richards and Borglin (2011).

## Towards achieving outcomes that matter to clients

The Wakefield *et al*. (2020) study serves to legitimate current IAPT practice. These authors are remiss in not pointing out that IAPT’s studies reveal no evidence of enduring loss of diagnostic status. As such, they display an indifference to what clients would regard as evidence of treatment making a real‐world difference. There is a pressing need for a publicly funded independent evaluation of IAPT’s work, one that appropriately takes into account the methodological rigour that has been refined in the last decade. Until such time, the burden of proof is on IAPT to provide credible evidence of effectiveness. Although the author’s own study Scott (2018) is not without its’ limitation (IAPT clients who had been PI litigants at some stage) it was at least independent, assessed outcome using a ‘gold standard’, and described client’s reported experiences of the service. There is also a need for an independent qualitative study of clients and clinician’s experiences of IAPT.

## Conflicts of interest

All authors declare no conflict of interest.
